# Crystal structure of a photobiologically active brominated angular pyran­ocoumarin: bromo-hy­droxy-seselin

**DOI:** 10.1107/S2056989017002808

**Published:** 2017-02-28

**Authors:** A. K. Bauri, Sabine Foro, A. F. M. Mustafizur Rahman

**Affiliations:** a Bio-Organic Division, Bhabha Atomic Research Centre, Trombay, Mumbai 400 085, India; b Institute of Materials Science, Darmstadt University of Technology, Alarich-Weiss-Strasse 2, D-64287 Darmstadt, Germany; c Department of Applied Chemistry & Chemical Engineering, University of Dhaka, Dhaka, Dhaka-1000, Bangladesh

**Keywords:** crystal structure, seselin, bromination, hydrogen bonding, offset π–π inter­actions

## Abstract

The title compound, C_14_H_13_BrO_3_ [systematic name: *rac*-(9*S*,10*R*)-9-bromo-10-hy­droxy-8,8-dimethyl-9,10-di­hydro-2*H*,8*H*-pyrano[2,3-*f*]chromen-2-one], is a substituted pyran­ocoumarin, obtained by bromination of seselin [8,8-dimethyl-2*H*,8*H*-pyrano[2,3-*f*]chromen-2-one], which was isolated from the Indian herb *Trachyspermum stictocarpum* (Aajmod). The pyrano ring has a distorted half-chair conformation and its mean plane is inclined to the coumarin mean plane by 1.6 (2)°. In the crystal, mol­ecules are linked by pairs of O—H⋯O hydrogen bonds, forming inversion dimers with an *R* ^2^_2_(16) ring motif. The dimers stack along the *a*-axis direction and are linked by offset π–π inter­actions, forming columns [inter­centroid distance = 3.514 (4) Å].

## Abstract 2

The title compound, is a brominated product of seselin [8,8-dimethyl-2*H*,8*H*-pyrano[2,3-*f*]chromen-2-one], a photo biologically active compound.

## Chemical context   

**Figure 1:**
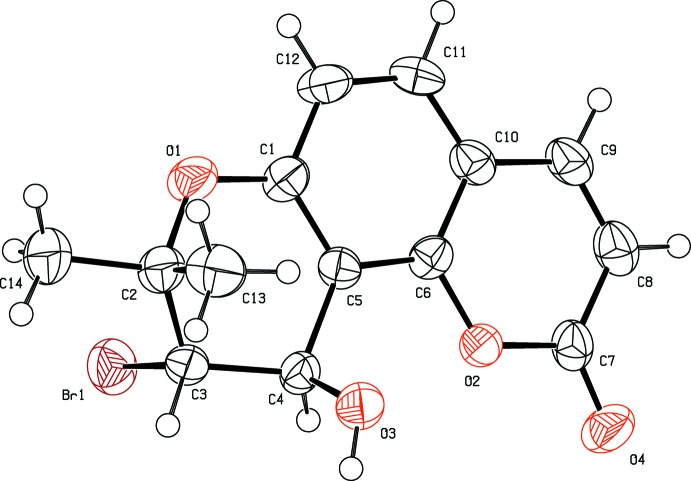
The mol­ecular structure of the title compound, with the atom labelling and displacement ellipsoids drawn at the 50% probability level

The title compound, *rac*-(9*S*,10*R*)-9-bromo-10-hy­droxy-8,8-dimethyl-9,10-di­hydro-2*H*,8*H*-pyrano[2,3-*f*]chromen-2-one, is a substituted product of the angular pyran­ocoumarin seselin, with a bromine atom and a hy­droxy group at the asymmetric carbon atoms C3 and C4 in the pyrano ring (see Fig. 1 ▸). This class of pyran­ocoumarins have absorption bands in the near UV region due to the presence of an extended conjugated enone system and exhibit photomutagenic (Appendino *et al.*, 2004 ▸) and photocarcinogenic properties, binding with purin bases of DNA in living cells to yield photoadducts (Filomena *et al.*, 2009 ▸). Based on this property, these compounds are employed to treat numerous inflammatory skin diseases such as atopic dermatitis and pigment disorders such as vitiligo and psoriasis on exposure to ultraviolet (UV) radiation in photodynamic therapy (PDT). As a result of their strong ability to absorb UV radiation, this class of mol­ecules are also utilized as photoprotective agents to prevent the absorption of harmful UV radiation by the skin in the form of a variety of sun-screening lotions, widely used in dermatological applications in the cosmetic and pharmaceutical industries (Chen *et al.*, 2007 ▸, 2009 ▸). In addition, *in vitro* anti­proliferative activity and *in vivo* phototoxicity of the parent mol­ecule has been reported against numerous cancer cell lines, including HL60, A431 (Conconi *et al.*, 1998 ▸). These classes of coumarins have been used successfully in combination with ultraviolet irradiation to treat psoriasis and vitiligo and have been found to inhibit proliferation in human hepatocellular carcinoma cell lines (March *et al.*, 1993 ▸). Experimental results revealed that their phototoxicity is exerted *via* Diels–Alder reactions, binding to the double bond of a purin base of DNA in living cells with double bonds of the coumarin, to yield mono- and di-adducts (Conforti *et al.*, 2009 ▸). Recently, this type of mol­ecule has been combined with a porphyrin to obtain a scaffold-type macromolecule and employed to study of its inter­action (host–guest inter­action) with fullerenes, such as C_60_ and C_70_ in supra­molecular chemistry (Banerjee *et al.*, 2014 ▸; Ghosh *et al.*, 2014 ▸). The mol­ecular tweezers containing a coumarin moiety showed better quantum yield and fluorescence absorption due to the presence of the extended conjugated enone of pyran­ocoumarin. As part of our studies in this area, we now describe the synthesis and structure of the title compound.
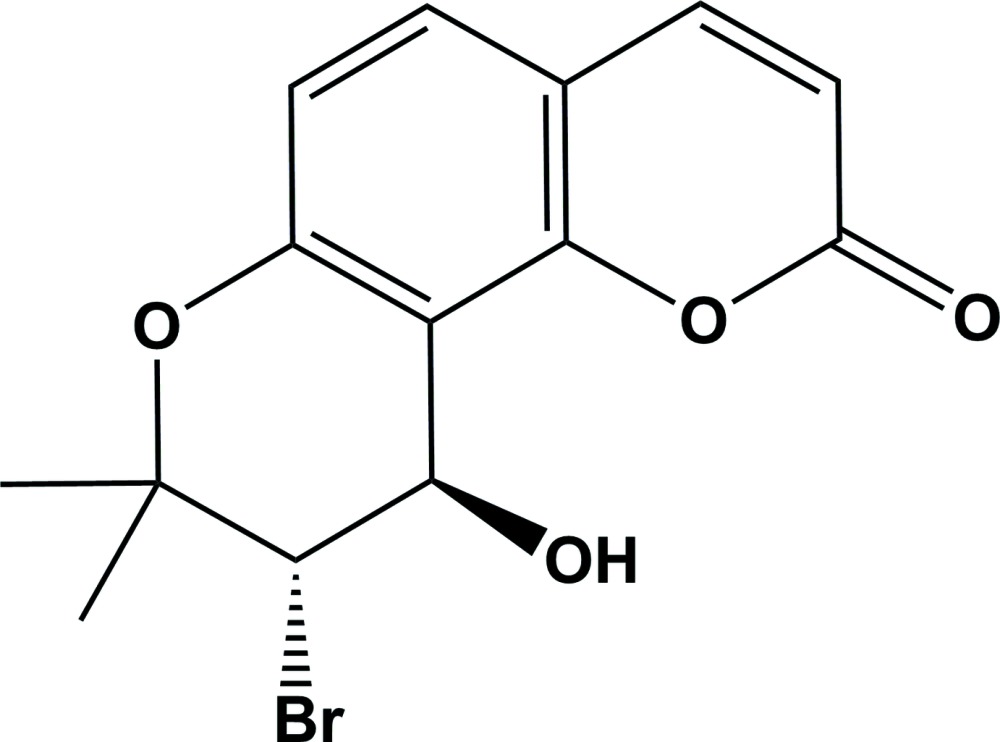


## Structural commentary   

The title compound, Fig. 1 ▸, belongs to a class of naturally occurring pyran­ocoumarins, known as psoralenes. It is an angular isomer of the substituted pyran­ocoumarin seselin [8,8-dimethyl-2*H*,8*H*-pyrano[2,3-*f*]chromen-2-one], whose crystal structure has been reported (Kato, 1970 ▸; Bauri *et al.*, 2006 ▸). It is composed of three different ring systems, *viz.* benzene, pyrone and pyrano, with (CH_3_)_2_, Br and OH substituents located at the C2, C3 and C4 positions, respectively, see Fig. 1 ▸. The C5—C6—C10—C9 and O2—C6—C10—C11 torsion angles are almost the same, *viz.* 178.6 (6) and 178.3 (5)°, respectively, indicating that these rings are almost coplanar. The pyrano ring (O1/C1–C5) has a distorted half-chair conformation [puckering parameters: amplitude (*Q*) = 0.443 (7) Å, θ = 132.7 (9)°, φ = 91.7 (11)°], probably due to ring flexibility and the presence of the substituents. Its mean plane is inclined to the mean plane of the coumarin ring by 1.6 (2)°. There are two asymmetric centres at positions C3 and C4 in the mol­ecule (Fig. 1 ▸). The present study of the title racemic compound revealed that the relative configuration of atoms C3 and C4 to be *S* and *R*, respectively.

## Supra­molecular features   

**Figure 2:**
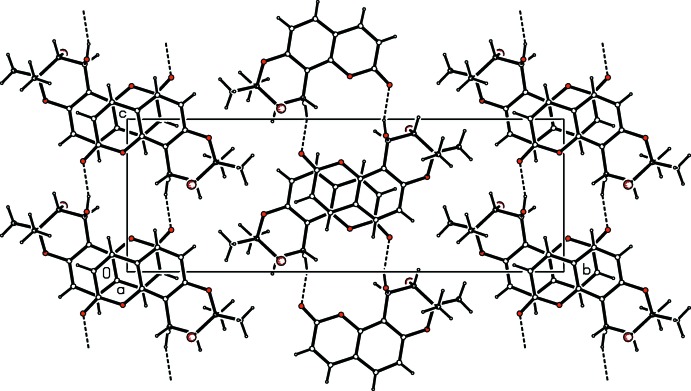
A view along the *a* axis of the crystal packing of the title compound, with hydrogen bonds shown as dashed lines (see Table 1 ▸).

In the crystal, mol­ecules are linked by pairs of O—H⋯O hydrogen bonds, forming inversion dimers with an 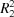
(16) ring motif (Table 1 ▸ and Fig. 2 ▸). The dimers stack along the *a*-axis direction and are linked by offset π–π inter­actions, forming columns [*Cg*2⋯*Cg*2(−*x* + 1, −*y*, −*z* + 2) = 3.514 (4) Å, inter­planar distance = 3.422 (3) Å, slippage = 0.798 Å; *Cg*2 is the centroid of the O2/C6–C10 ring].

## Database survey   

A search of the Cambridge Structural Database (CSD, Version 5.38, last update November 2016; Groom *et al.*, 2016 ▸) gave more than 25 hits for the pyran­ocoumarin structure. They include two reports of the crystal structure of seselin [CSD refcodes AMYROL (Kato, 1970 ▸) and AMYROL01 (Bauri *et al.*, 2006 ▸)], and a number of structures with various substituents at the C3 and C4 atoms; many of which are natural products.

## Synthesis and crystallization   

The compound seselin was isolated as a colourless crystalline solid from the methanol extract of *T. stictocarpum* (in local dialect known as *Aajmod*) by means of column chromatography over SiO_2_ gel, by gradient elution with a mixture of a binary solvent system of hexane and ethyl acetate. It was purified by reverse-phase high-pressure liquid chromatography followed by crystallization to yield a colourless solid. This compound was then brominated using NBS in aqueous tetra­hydro­furan (THF) in a 1:1 ratio at room temperature with continuous mechanical stirring over a period of 12 h. The reaction was quenched with ice-cold water and extracted with diethyl ether to yield the crude product. This was then purified by column chromatography over SiO_2_ with gradient solvent elution to yield the title compound. Colourless rod-like crystals were obtained after recrystallization three times from ethyl acetate:hexane (1:4) solution at room temperature.

## Refinement   

Crystal data, data collection and structure refinement details are summarized in Table 2 ▸. The hydroxyl H atom was located in a difference Fourier map and refined with *U*_iso_(H) = 1.2*U*_eq_(O). The C-bound H atoms were included in calculated positions and treated as riding atoms: C—H = 0.93–0.98 Å with *U*_iso_(H) = 1.2*U*_eq_(C).

## Supplementary Material

Crystal structure: contains datablock(s) I, Global. DOI: 10.1107/S2056989017002808/su5353sup1.cif

Structure factors: contains datablock(s) I. DOI: 10.1107/S2056989017002808/su5353Isup2.hkl

CCDC reference: 1533622

Additional supporting information:  crystallographic information; 3D view; checkCIF report

## Figures and Tables

**Table 1:** Hydrogen-bond geometry (Å, °)

*D*—H⋯*A*	*D*—H	H⋯*A*	*D*⋯*A*	*D*—H⋯*A*
O3—H3*O*⋯O4^i^	0.81 (2)	1.95 (3)	2.734 (7)	162 (8)

Symmetry code: (i) 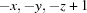.

**Table 2:** Experimental details

Crystal data	
Chemical formula	C_14_H_13_BrO_4_
*M*_r_	325.15
Crystal system, space group	Monoclinic, *P*2_1_/*n*
Temperature (K)	299
*a*, *b*, *c* (Å)	6.9573 (6), 23.465 (2), 8.3435 (7)
β (°)	100.79 (1)
*V* (Å^3^)	1338.0 (2)
*Z*	4
Radiation type	Mo *K*α
μ (mm^−1^)	3.08
Crystal size (mm)	0.44 × 0.20 × 0.16

Data collection	
Diffractometer	Oxford Diffraction Xcalibur Sapphire CCD detector
Absorption correction	Multi-scan (*CrysAlis RED*; Oxford Diffraction, 2009 ▸)
*T*_min_, *T*_max_	0.344, 0.639
No. of measured, independent and observed [*I* > 2σ(*I*)] reflections	4521, 2392, 2063
*R*_int_	0.022
(sin θ/λ)_max_ (Å^−1^)	0.602

Refinement	
*R*[*F* ^2^ > 2σ(*F* ^2^)], *wR*(*F* ^2^), *S*	0.063, 0.202, 1.12
No. of reflections	2392
No. of parameters	175
No. of restraints	1
H-atom treatment	H atoms treated by a mixture of independent and constrained refinement
Δρ_max_, Δρ_min_ (e Å^−3^)	1.25, −1.02

Computer programs: *CrysAlis CCD* and *CrysAlis RED* (Oxford Diffraction, 2009 ▸), *SHELXS97* and *SHELXL97* (Sheldrick, 2008 ▸) and *PLATON* (Spek, 2009 ▸).

## supplementary crystallographic information

### Crystal data

**Table:**

C_14_H_13_BrO_4_	*F*(000) = 656
*M**_r_* = 325.15	*D*_x_ = 1.614 Mg m^−^^3^
Monoclinic, *P*2_1_/*n*	Mo *K*α radiation, λ = 0.71073 Å
Hall symbol: -P 2yn	Cell parameters from 3332 reflections
*a* = 6.9573 (6) Å	θ = 2.5–27.4°
*b* = 23.465 (2) Å	µ = 3.08 mm^−^^1^
*c* = 8.3435 (7) Å	*T* = 299 K
β = 100.79 (1)°	Rod, colourless
*V* = 1338.0 (2) Å^3^	0.44 × 0.20 × 0.16 mm
*Z* = 4	

### Data collection

**Table:**

Oxford Diffraction Xcalibur Sapphire CCD detector diffractometer	2392 independent reflections
Radiation source: fine-focus sealed tube	2063 reflections with *I* > 2σ(*I*)
Graphite monochromator	*R*_int_ = 0.022
Rotation method data acquisition using ω scans.	θ_max_ = 25.4°, θ_min_ = 2.6°
Absorption correction: multi-scan (CrysAlis RED; Oxford Diffraction, 2009)	*h* = −8→8
*T*_min_ = 0.344, *T*_max_ = 0.639	*k* = −20→28
4521 measured reflections	*l* = −10→6

### Refinement

**Table:**

Refinement on *F*^2^	Primary atom site location: structure-invariant direct methods
Least-squares matrix: full	Secondary atom site location: difference Fourier map
*R*[*F*^2^ > 2σ(*F*^2^)] = 0.063	Hydrogen site location: inferred from neighbouring sites
*wR*(*F*^2^) = 0.202	H atoms treated by a mixture of independent and constrained refinement
*S* = 1.12	*w* = 1/[σ^2^(*F*_o_^2^) + (0.0969*P*)^2^ + 6.1833*P*] where *P* = (*F*_o_^2^ + 2*F*_c_^2^)/3
2392 reflections	(Δ/σ)_max_ = 0.005
175 parameters	Δρ_max_ = 1.25 e Å^−^^3^
1 restraint	Δρ_min_ = −1.02 e Å^−^^3^

### Special details

**Table:**

Geometry. All esds (except the esd in the dihedral angle between two l.s. planes) are estimated using the full covariance matrix. The cell esds are taken into account individually in the estimation of esds in distances, angles and torsion angles; correlations between esds in cell parameters are only used when they are defined by crystal symmetry. An approximate (isotropic) treatment of cell esds is used for estimating esds involving l.s. planes.
Refinement. Refinement of F^2^ against ALL reflections. The weighted R-factor wR and goodness of fit S are based on F^2^, conventional R-factors R are based on F, with F set to zero for negative F^2^. The threshold expression of F^2^ &gt; 2sigma(F^2^) is used only for calculating R-factors(gt) etc. and is not relevant to the choice of reflections for refinement. R-factors based on F^2^ are statistically about twice as large as those based on F, and R- factors based on ALL data will be even larger.

### Fractional atomic coordinates and isotropic or equivalent isotropic displacement parameters (Å^2^)

**Table:**

	*x*	*y*	*z*	*U*_iso_*/*U*_eq_	
C1	0.2945 (9)	0.1326 (3)	0.9293 (8)	0.0385 (14)	
C2	0.2080 (10)	0.2030 (3)	0.7183 (8)	0.0439 (15)	
C3	0.2309 (9)	0.1564 (3)	0.5932 (8)	0.0370 (14)	
H3	0.1538	0.1673	0.4870	0.044*	
C4	0.1631 (8)	0.0972 (3)	0.6392 (7)	0.0337 (13)	
H4	0.2193	0.0678	0.5787	0.040*	
C5	0.2295 (8)	0.0878 (3)	0.8222 (7)	0.0323 (13)	
C6	0.2285 (8)	0.0340 (3)	0.8890 (8)	0.0327 (13)	
C7	0.1645 (9)	−0.0657 (3)	0.8329 (8)	0.0392 (15)	
C8	0.2141 (10)	−0.0764 (3)	1.0051 (9)	0.0450 (16)	
H8	0.2076	−0.1135	1.0430	0.054*	
C9	0.2692 (10)	−0.0345 (3)	1.1120 (8)	0.0415 (15)	
H9	0.3007	−0.0427	1.2229	0.050*	
C10	0.2804 (9)	0.0233 (3)	1.0572 (8)	0.0367 (14)	
C11	0.3418 (10)	0.0693 (3)	1.1591 (8)	0.0425 (15)	
H11	0.3787	0.0635	1.2709	0.051*	
C12	0.3486 (11)	0.1233 (3)	1.0959 (8)	0.0462 (16)	
H12	0.3897	0.1537	1.1653	0.055*	
C13	−0.0073 (12)	0.2122 (3)	0.7295 (10)	0.0554 (19)	
H13A	−0.0167	0.2418	0.8069	0.066*	
H13B	−0.0602	0.1775	0.7641	0.066*	
H13C	−0.0797	0.2230	0.6244	0.066*	
C14	0.2986 (14)	0.2596 (3)	0.6815 (11)	0.062 (2)	
H14A	0.4356	0.2543	0.6823	0.075*	
H14B	0.2819	0.2871	0.7629	0.075*	
H14C	0.2353	0.2730	0.5760	0.075*	
O1	0.3137 (7)	0.18705 (19)	0.8778 (6)	0.0473 (12)	
O2	0.1716 (6)	−0.01003 (17)	0.7824 (5)	0.0366 (10)	
O3	−0.0446 (7)	0.0926 (2)	0.6070 (6)	0.0455 (11)	
H3O	−0.078 (11)	0.089 (4)	0.510 (3)	0.055*	
O4	0.1163 (8)	−0.1010 (2)	0.7257 (6)	0.0562 (14)	
Br1	0.50492 (10)	0.14733 (3)	0.57070 (9)	0.0528 (3)	

### Atomic displacement parameters (Å^2^)

**Table:**

	*U*^11^	*U*^22^	*U*^33^	*U*^12^	*U*^13^	*U*^23^
C1	0.038 (3)	0.034 (3)	0.041 (4)	−0.001 (3)	0.000 (3)	−0.002 (3)
C2	0.057 (4)	0.031 (3)	0.041 (4)	−0.001 (3)	0.000 (3)	0.003 (3)
C3	0.039 (3)	0.038 (3)	0.028 (3)	−0.005 (2)	−0.009 (2)	0.008 (3)
C4	0.033 (3)	0.032 (3)	0.034 (3)	−0.005 (2)	0.000 (2)	0.003 (3)
C5	0.030 (3)	0.034 (3)	0.030 (3)	0.001 (2)	−0.001 (2)	0.006 (3)
C6	0.028 (3)	0.032 (3)	0.036 (3)	0.002 (2)	−0.001 (2)	0.001 (3)
C7	0.038 (3)	0.035 (3)	0.043 (4)	−0.001 (3)	0.004 (3)	0.011 (3)
C8	0.044 (4)	0.044 (4)	0.047 (4)	0.002 (3)	0.008 (3)	0.015 (3)
C9	0.044 (4)	0.045 (4)	0.034 (3)	0.004 (3)	0.004 (3)	0.012 (3)
C10	0.034 (3)	0.043 (4)	0.032 (3)	0.003 (3)	0.003 (2)	0.007 (3)
C11	0.048 (4)	0.053 (4)	0.024 (3)	0.006 (3)	0.000 (3)	0.001 (3)
C12	0.055 (4)	0.045 (4)	0.034 (4)	−0.001 (3)	−0.003 (3)	−0.010 (3)
C13	0.068 (5)	0.046 (4)	0.051 (4)	0.013 (4)	0.007 (4)	0.000 (3)
C14	0.087 (6)	0.041 (4)	0.056 (5)	−0.013 (4)	0.006 (4)	0.008 (4)
O1	0.063 (3)	0.033 (2)	0.041 (3)	−0.003 (2)	−0.005 (2)	0.000 (2)
O2	0.043 (2)	0.031 (2)	0.032 (2)	−0.0022 (18)	−0.0017 (18)	0.0054 (18)
O3	0.040 (2)	0.052 (3)	0.040 (3)	−0.008 (2)	−0.005 (2)	0.006 (2)
O4	0.079 (4)	0.033 (2)	0.048 (3)	−0.008 (2)	−0.011 (3)	0.000 (2)
Br1	0.0432 (4)	0.0606 (5)	0.0542 (5)	−0.0072 (3)	0.0085 (3)	0.0069 (4)

### Geometric parameters (Å, º)

**Table:**

C1—O1	1.362 (8)	C7—O2	1.376 (7)
C1—C12	1.388 (10)	C7—C8	1.435 (10)
C1—C5	1.399 (9)	C8—C9	1.336 (10)
C2—O1	1.444 (8)	C8—H8	0.9300
C2—C14	1.526 (9)	C9—C10	1.438 (9)
C2—C13	1.533 (11)	C9—H9	0.9300
C2—C3	1.540 (9)	C10—C11	1.391 (9)
C3—C4	1.538 (8)	C11—C12	1.375 (10)
C3—Br1	1.962 (7)	C11—H11	0.9300
C3—H3	0.9800	C12—H12	0.9300
C4—O3	1.424 (7)	C13—H13A	0.9600
C4—C5	1.526 (8)	C13—H13B	0.9600
C4—H4	0.9800	C13—H13C	0.9600
C5—C6	1.381 (8)	C14—H14A	0.9600
C6—O2	1.372 (7)	C14—H14B	0.9600
C6—C10	1.404 (9)	C14—H14C	0.9600
C7—O4	1.220 (8)	O3—H3O	0.81 (2)
			
O1—C1—C12	116.1 (6)	C9—C8—C7	121.6 (6)
O1—C1—C5	122.9 (6)	C9—C8—H8	119.2
C12—C1—C5	121.0 (6)	C7—C8—H8	119.2
O1—C2—C14	104.7 (6)	C8—C9—C10	120.6 (6)
O1—C2—C13	108.3 (6)	C8—C9—H9	119.7
C14—C2—C13	109.6 (6)	C10—C9—H9	119.7
O1—C2—C3	109.9 (5)	C11—C10—C6	117.7 (6)
C14—C2—C3	112.5 (6)	C11—C10—C9	124.5 (6)
C13—C2—C3	111.5 (6)	C6—C10—C9	117.8 (6)
C4—C3—C2	113.3 (5)	C12—C11—C10	120.5 (6)
C4—C3—Br1	105.9 (4)	C12—C11—H11	119.7
C2—C3—Br1	111.6 (4)	C10—C11—H11	119.7
C4—C3—H3	108.6	C11—C12—C1	120.5 (6)
C2—C3—H3	108.6	C11—C12—H12	119.7
Br1—C3—H3	108.6	C1—C12—H12	119.7
O3—C4—C5	106.5 (5)	C2—C13—H13A	109.5
O3—C4—C3	111.7 (5)	C2—C13—H13B	109.5
C5—C4—C3	109.4 (5)	H13A—C13—H13B	109.5
O3—C4—H4	109.7	C2—C13—H13C	109.5
C5—C4—H4	109.7	H13A—C13—H13C	109.5
C3—C4—H4	109.7	H13B—C13—H13C	109.5
C6—C5—C1	117.1 (5)	C2—C14—H14A	109.5
C6—C5—C4	120.9 (5)	C2—C14—H14B	109.5
C1—C5—C4	122.0 (5)	H14A—C14—H14B	109.5
O2—C6—C5	116.7 (5)	C2—C14—H14C	109.5
O2—C6—C10	120.2 (5)	H14A—C14—H14C	109.5
C5—C6—C10	123.1 (6)	H14B—C14—H14C	109.5
O4—C7—O2	116.1 (6)	C1—O1—C2	118.1 (5)
O4—C7—C8	126.6 (6)	C6—O2—C7	122.5 (5)
O2—C7—C8	117.3 (6)	C4—O3—H3O	107 (6)
			
O1—C2—C3—C4	56.8 (7)	O2—C7—C8—C9	−1.5 (10)
C14—C2—C3—C4	173.0 (6)	C7—C8—C9—C10	0.1 (10)
C13—C2—C3—C4	−63.4 (7)	O2—C6—C10—C11	178.3 (5)
O1—C2—C3—Br1	−62.7 (6)	C5—C6—C10—C11	−2.3 (9)
C14—C2—C3—Br1	53.5 (7)	O2—C6—C10—C9	−0.7 (9)
C13—C2—C3—Br1	177.1 (5)	C5—C6—C10—C9	178.6 (6)
C2—C3—C4—O3	76.8 (6)	C8—C9—C10—C11	−178.0 (7)
Br1—C3—C4—O3	−160.5 (4)	C8—C9—C10—C6	1.0 (9)
C2—C3—C4—C5	−40.8 (7)	C6—C10—C11—C12	0.8 (10)
Br1—C3—C4—C5	81.9 (5)	C9—C10—C11—C12	179.8 (7)
O1—C1—C5—C6	176.0 (6)	C10—C11—C12—C1	−0.1 (11)
C12—C1—C5—C6	−2.3 (9)	O1—C1—C12—C11	−177.4 (6)
O1—C1—C5—C4	−3.7 (9)	C5—C1—C12—C11	0.9 (11)
C12—C1—C5—C4	178.1 (6)	C12—C1—O1—C2	−161.8 (6)
O3—C4—C5—C6	74.4 (7)	C5—C1—O1—C2	19.9 (9)
C3—C4—C5—C6	−164.7 (5)	C14—C2—O1—C1	−166.3 (6)
O3—C4—C5—C1	−105.9 (6)	C13—C2—O1—C1	76.9 (7)
C3—C4—C5—C1	14.9 (8)	C3—C2—O1—C1	−45.2 (8)
C1—C5—C6—O2	−177.6 (5)	C5—C6—O2—C7	179.9 (5)
C4—C5—C6—O2	2.1 (8)	C10—C6—O2—C7	−0.7 (8)
C1—C5—C6—C10	3.1 (9)	O4—C7—O2—C6	−178.2 (6)
C4—C5—C6—C10	−177.3 (6)	C8—C7—O2—C6	1.8 (8)
O4—C7—C8—C9	178.5 (7)		

### Hydrogen-bond geometry (Å, º)

**Table:**

*D*—H···*A*	*D*—H	H···*A*	*D*···*A*	*D*—H···*A*
O3—H3*O*···O4^i^	0.81 (2)	1.95 (3)	2.734 (7)	162 (8)

Symmetry code: (i) −*x*, −*y*, −*z*+1.

## References

[bb1] Appendino, G., Bianchi, F., Bader, A., Campagnuolo, C., Fattorusso, E., Taglialatela-Scafati, O., Blanco-Molina, M., Macho, A., Fiebich, B. L., Bremner, P., Heinrich, M., Ballero, M. & Muñoz, E. (2004). *J. Nat. Prod.* **67**, 532–536. 10.1021/np0340652 15104479

[bb2] Banerjee, S., Ghosh, B. K., Bauri, A. K. & Bhattacharya, S. (2014). *J Spectrosc. Dyn.* **4**, 29–34.

[bb3] Bauri, A. K., Foro, S., Lindner, H.-J. & Nayak, S. K. (2006). *Acta Cryst.* E**62**, o1340–o1341.

[bb4] Chen, Y., Fan, G., Zhang, Q., Wu, H. & Wu, Y. (2007). *J. Pharm. Biomed. Anal.* **43**, 926–936. 10.1016/j.jpba.2006.09.015 17046189

[bb5] Chen, D., Wang, J., Jiang, Y., Zhou, T., Fan, G. & Wu, Y. (2009). *J. Pharm. Biomed. Anal.* **50**, 695–702. 10.1016/j.jpba.2009.05.026 19608371

[bb6] Conconi, M. T., Montesi, F. & Parnigotto, P. P. (1998). *Basic Clin. Pharmacol. Toxicol.* **82**, 193–198. 10.1111/j.1600-0773.1998.tb01424.x 9584334

[bb7] Conforti, F., Marrelli, M., Menichini, F., Bonesi, M., Statti, G., Provenzano, E. & Menichini, F. (2009). *Curr. Drug Ther.* **4**, 38–58.

[bb8] Ghosh, B. K., Bauri, A. K., Bhattacharya, S. & Banerjee, S. (2014). *Spectrochim. Acta Part A*, **125**, 90–98. 10.1016/j.saa.2013.11.059 24531105

[bb9] Groom, C. R., Bruno, I. J., Lightfoot, M. P. & Ward, S. C. (2016). *Acta Cryst.* B**72**, 171–179. 10.1107/S2052520616003954 PMC4822653 27048719

[bb10] Kato, K. (1970). *Acta Cryst.* B**26**, 2022–2029.

[bb11] March, K. L., Patton, B. L., Wilensky, R. L. & Hathaway, D. R. (1993). *Circulation*, **87**, 184–191. 10.1161/01.cir.87.1.184 8419006

[bb12] Oxford Diffraction (2009). *CrysAlis CCD* & *CrysAlis RED*. Oxford Diffraction Ltd., Abingdon, England.

[bb13] Sheldrick, G. M. (2008). *Acta Cryst.* A**64**, 112–122. 10.1107/S0108767307043930 18156677

[bb14] Spek, A. L. (2009). *Acta Cryst.* D**65**, 148–155. 10.1107/S090744490804362X PMC2631630 19171970

